# Supplementary material for: Maternal perception of masking in children as a preventive strategy for COVID-19 in Nigeria: A multicentre study
Source: PLoS One. 2020 Nov 19;15(11):e0242650. doi: 10.1371/journal.pone.0242650 (PMC7676872; doi:10.1371/journal.pone.0242650)
Supplement: S2 File — (DOCX) [file pone.0242650.s002.docx]

This questionnaire is designed to help us understand better the effect of Corona virus pandemic on mothers’ health seeking behaviour. This will help us to give better counsel to the mothers.

All information given to us will be treated as strictly confidential and will not be made available to any other party.

Your co-operation is highly appreciated. Please help us give your answers as accurately as possible. Thank you.

**A.1.NAME(OPTIONAL)……………………………………………………………………..**

**2.HOSPITAL……………………………………………………………………………………**

**3.STATE OF ORIGIN……………………………..4.TRIBE…………………………………**

**5.MARITAL STATUS**: 1 married 2 Single 3 Divorced 4 widowed 5 separated

**6..OCCUPATION…………………………………………………………………………………………**

**7.HIGHEST EDUCATIONAL LEVEL……………..**

**8.AGE NEXT BIRTHDAY……………………**

**9.HUSBANDS’OCCUPATION…………………………………………………………………………**

**10.HUSBANDS’ HIGHEST EDUCATIONAL LEVEL………………………………………………**

**11.HUSBAND’S AGE NEXT BIRTHDAY…………………………………………………………**

**12.PARITY…………………13.AGE OF LAST CHILD…………………………………………..**

**14.NUMBER OF CHILDREN………………………………………………………………**

**B**. **PLEASE ANSWER THESE QUESTIONS BELOW AS ACCURATELY AS YOU CAN.**

**1. At what age did your child present to the facility** [1] <8 days [ 2] 8-1 year [3 ] I year-5 years [4 ] 6 years to 12 years [ 5] Beyond 12 years

2. **Why did your child present to the hospital** [1] had Fever [2] Charm from village

[3] diarrhoea [4] not well develped [5] Cough [6] Convulsion [7] Don’t know

Duration of illness:……………

3**. What is the sex of your child** [a] Male [b] Female

**4. What did you do when your child became sick at this period of Corona?**

[ 1] saw a nurse [2] Stayed at home [3] Visited a herbalist [ 4] Self medication [5] Pharmacy [6] Chemist shop [7] Herbal medication [8] Don’t Know [9] Social media advice

**5. If you stayed at home ,why?**  [1] Fear of getting Corona [ 2] No money [3]prefer self medication [4] No preferences

**6. Can somebody who is not sick spread Corona virus? YES/NO**

**7. Can childen contact Corona Virus?**

[ 1] Yes [2] No [3] Not sure [4]Dont Know

**8. IF YES, do they have severe forms?** [ 1] Yes [2] No [3] Not sure [4]Dont Know

9**. For the past 6 Months, how many times have you come to the Hospital?**

1] no visit [2] once [ 3] 2 times [4]3 times [5]> 3 times

10. **What is the Current health status of those that attended to you in the hospital**

1] Poor [2 ] Average [ 3] Good [4]Better [5] Excellent

11. **What is the attitude of those that attended to you in the hospital**

1] Friendly [2 ] Rejection [ 3] Hostile [4] Abandonment [5] Excellent care

12. **What is your Perception of most common acute illness in the area**

[1] Corona virus [2] Malaria [3] Diarrhoea [4] Pneumonia 5] Don’t know

13. **Which health facilty will you choose if your child is ill this Corona period**

[1] Government [2] Private [3] I will stay at home 4]Don’t know

14. **How did you reach the health facility**

[1] Walking [2] Public transport [3] Bicycle [4] Private car

15. **Time taken to reach the nearest health facility**

[1] <15 mins [2] 15-30Mins [3]30-60 Minutes [4]> 60 minutes

16 **Time gap between visit to the health facility and the onset of symptoms**

[1] 1 Day [2] 2 Days [3]3 Days [4] > 3 days

17. **Did you spend more money in this present hospital visit than before when there were no COVID**

[ 1] Yes [2] No [3] Not sure [4]Dont Know

18. Covid 19 prevention guideline available: Tick as applicable

Use of face mask for all (staff and patients), hand washing, social distancing, cough hygiene

19. Does your child wear face mask? YES/NO

20. If NO, WHY?

Cries when mask is on, pulls it off, has difficulty breathing when mask is on,any OTHER reason----------------
