# Supplementary material for: Maternal perception of masking in children as a preventive strategy for COVID-19 in Nigeria: A multicentre study
Source: PLoS One. 2020 Nov 19;15(11):e0242650. doi: 10.1371/journal.pone.0242650 (PMC7676872; doi:10.1371/journal.pone.0242650)
Supplement: Attachment — Submitted filename: REVIEW for PLOS ONE MS.docx [file pone.0242650.s003.docx]

REVIEWER’S REPORT ON SUBMITTED MANUSCRIPT

MANUSCRIPT ID- PONE-D-20-19085

Title- Maternal factors and predictors of wearing face mask in children, in the prevention of COVID-19. A multicentre study

General comments.

The authors of this manuscript essentially conducted a KAP study on mothers regarding the wearing of facemask in their children. It was a timely multi-centre, cross-sectional descriptive study in a sub-Saharan African setting which is not spared by the global COVID-19 pandemic. Thus, establishing the KAP on this important preventive strategy of the pandemic is relevant. However, there are some major and minor concerns about this manuscript.

Major concerns

1. Under the Abstract and title, the authors need to note these flaws: The title requires modification for clarity. It is not reflective of the content of the manuscript. The background did not provide the research gap to be filled. The study aim was not explicit enough (it was like a repetition of the study title). For methodology, pertinent elements like sample size, study instrument and how it was administered were missing. Reportage of the results should be improved. The conclusion is not in sync with the results
2. Introduction- The first sentence which reads- **‘Corona Virus infection (COVID 19) has now attained community infection and is now very common in families’**- appears rather ambiguous. I think corona virus infection is not synonymous with COVID-19 (the latter is the disease caused by the former). Do the authors mean that the pandemic is now at the level of community transmission? Although the authors tried to highlight what is already known about the topic and the research question, the aim of the study as stated here (‘ascertaining the perception of mothers on the use of face mask in children in this COVID era’) is different from the aim as stated under the Abstract.
3. Methods- The authors revealed the study instrument here as structured self-administered questionnaires. A concise detail of the questionnaire items would have enlightened the reader. Would all the respondents have understood the items? An interviewer-administered questionnaire could have been a better approach.
4. Results- The ‘practice’ aspect of this study namely ‘the wearing of face mask in children’ was based on proxy-report and not observational. Thus, the information provided by these mothers on the proportion of their children who did wear face mask may not be reliable. This underscores the fact that an observational study of mother-child dyads would have been better. For instance, the authors reported that children aged 8 days to 1 year wore face masks. The practice is even contradictory to the reported WHO recommendation in children. The authors reported some socio-demographic variables as predictors of the practice of wearing face mask in children. Curiously, they were silent on the role of knowledge and perception. Maternal knowledge and perception ought to influence their practice of using face mask on their children
5. Discussion- The content and flow of this section should be improved. The authors did not highlight any limitations of the current study. The conclusion was not precise enough but was too concise to have conveyed the message of the study.

Minor

1. The manuscript prose needs improvement
2. The language requires professional editing
