# Supplementary material for: Maternal perception of masking in children as a preventive strategy for COVID-19 in Nigeria: A multicentre study
Source: PLoS One. 2020 Nov 19;15(11):e0242650. doi: 10.1371/journal.pone.0242650 (PMC7676872; doi:10.1371/journal.pone.0242650)
Supplement: Attachment — Submitted filename: PONIT BY POINT FACE MASK.docx [file pone.0242650.s004.docx]

Department of pediatrics,

College of Medicine,

University of Nigeria Enugu Campus (UNEC),

University of Nigeria Teaching Hospital,

PMB 01129,

Postal Code 400001, ENUGU, NIGERIA.

20^th^ August, 2020

Editor-in-Chief,

Plos One

**Dear Sir,**

**MANUSCRIPT CORRECTION: RESPONSES TO EDITOR’S COMMENTS**

The authors are grateful to you, all the members of the Editorial Board and the

reviewers for giving us the opportunity to make corrections on our manuscript titled”

**Maternal factors and predictors of wearing face mask on children, in the prevention of COVID 19. A multicentre study.”**

Please find below point-by-point corrections made, based on the editor’s comments:

**Editors comment**

1. Please ensure that your manuscript meets PLOS ONE's style requirements, including those for file naming.

**Authors Response**

**We have done this.**

**Editors comment**

2. Please amend your current ethics statement to address the following concerns:  
a) Did participants provide their written or verbal informed consent to participate in this study?
**Authors Response**

**We have added consent to participate section. See page 8 line 168-171**

**Editors comment**

b) If consent was verbal, please explain i) why written consent was not obtained, ii) how you documented participant consent, and iii) whether the ethics committees/IRB approved this consent procedure.

**Authors Response**

**We have explained why we obtained verbal consent. See page 8 line 172-175**

**Editors comment**

3. Please include additional information regarding the survey or questionnaire used in the study and ensure that you have provided sufficient details that others could replicate the analyses.

**Authors Response**

**We have included additional details with respect to questionnaire used in the study. See page 7 line 148-150**

**Editors comment**

For instance, if you developed a questionnaire as part of this study and it is not under a copyright more restrictive than CC-BY, please include a copy, in both the original language and English, as Supporting Information.

**Authors Response**

**We have added a copy of the questionnaire as Supporting Information.**

**Editors comment**

4. Please state whether you validated the questionnaire prior to testing on study participants. Please provide details regarding the validation group within the methods section.

**Authors Response**

**A pretested, semi-structured, interviewer-administered questionnaire was used. See page 7 line 148-150**

**Editors comment**

5. In your Data Availability statement, you have not specified where the minimal data set underlying the results described in your manuscript can be found. PLOS defines a study's minimal data set as the underlying data used to reach the conclusions drawn in the manuscript and any additional data required to replicate the reported study findings in their entirety. All PLOS journals require that the minimal data set be made fully available. For more information about our data policy, please see <http://journals.plos.org/plosone/s/data-availability>.

**Editors comment**

 7. Please include your tables as part of your main manuscript and

**Authors Response**

**We have included tables in the manuscripts and we have also removed the individual files. See page 9-13**

**Editors comment**

Please note that supplementary tables should be uploaded as separate "supporting information" files

**Authors Response**

**We have taken note of that**

**Reviewer’s comments**

Reviewer #1: Thank you for raising the important issue of children wearing a face mask and the maternal predictive factors. This is an important and very timely issues.

**Authors response**

**Thanks for the commendation**

**Reviewer’s comments**

Authors have done a good analysis of literature but what is lacking is the policy guidelines in Nigeria. based on WHO or other recommendations- what has the government mandated and disseminated. this is not described. Most countries utilize the global guidance from WHO but contextualize it.

**Authors response**

**We have added the WHO recommendations of wearing face mask in introduction and discussion. See page 5 line 95-102. We have also added the WHO recommendations in the Nigerian context. See page 5 line 97-102**

**Reviewer’s comments**

Authors claim that this study will establish the need to wear a mask. While the authors have quoted from the literature about the need to wear a mask, i don't think the analysis lends itself to convincing people on the need t wear a mask.

**Authors response**

**This statement has been expunged**

**Reviewer’s comments**

The question that authors are seeking to answer should be further clarified. Is it the proportion of women who have the knowledge that face mask is essential for children? You write- proportion of mothers who have knowledge of child wearing of face mask. Please describe in detail how you assess the knowledge and the perception.

**Authors response**

**The focus is use of facemask by the child. The mothers’ perception of COVID-19 has been included. See table 2 and 3**

**Reviewer’s comments**

In the statistical analysis - did you look for variation in young mothers by educational status and also for those above 35 by educational status? could be highly correlated. The age and education status Do you see the same predictive factors? It would have been interesting to see f the wearing of the masks by the mothers themselves also had the similar pattern.. Interesting to see that tertiary education of the father has the opposite effect on wearing a mask by the child. Could the authors explain why?
There is no study limitations mentioned. Please add. in addition- please strengthen the conclusion and what the authors would like to see change as a result of this study. Please add some recommendations for policy and programs

**Authors response**

**Information on wearing of facemask by mothers was not obtained. (This is now regarded as a limitation of the study). The perception of COVID-19 by the mothers was the focus for the mothers. In determining the factors affecting child use of facemask, the age of the mothers was categorized into three, <30 years, 30-34 years and 35 years and above. The educational attainment of the mothers and fathers of the children was categorized into tertiary education and secondary education and less.**

**We have explained why tertiary education of the father has the opposite effect on wearing a mask by the child. We have added limitation to the study, broadened the conclusion and we have mentioned recommendation for policies and program. See page 16 line 313-322**

**Reviewer’s 2 comments**

Reviewer #2: General comments.
The authors of this manuscript essentially conducted a KAP study on mothers regarding the wearing of facemask in their children. It was a timely multi-centre, cross-sectional descriptive study in a sub-Saharan African setting which is not spared by the global COVID-19 pandemic. Thus, establishing the KAP on this important preventive strategy of the pandemic is relevant. However, there are some major and minor concerns about this manuscript.

**Authors response**

**Thanks for the commendation**

**Reviewer’s 2 comments**

Major concerns
1. Under the Abstract and title, the authors need to note these flaws: The title requires modification for clarity. It is not reflective of the content of the manuscript. The background did not provide the research gap to be filled. The study aim was not explicit enough (it was like a repetition of the study title). For methodology, pertinent elements like sample size, study instrument and how it was administered were missing. Reportage of the results should be improved. The conclusion is not in sync with the results
**Authors response**

**We have snagged the title and provided the research gap in the background and the study aim has been expanded. We also added sample size, sample technique, study instrument to the study. The conclusion has been expanded. We have rewritten the reportage of the results. However, these modifications blew the abstract to more than the accepted 250 words format. See title and abstract**

**Reviewer’s 2 comments**

2. Introduction- The first sentence which reads- ‘Corona Virus infection (COVID 19) has now attained community infection and is now very common in families’- appears rather ambiguous. I think corona virus infection is not synonymous with COVID-19 (the latter is the disease caused by the former). Do the authors mean that the pandemic is now at the level of community transmission? Although the authors tried to highlight what is already known about the topic and the research question, the aim of the study as stated here (‘ascertaining the perception of mothers on the use of face mask in children in this COVID era’) is different from the aim as stated under the Abstract.

**Authors response**

**We have deleted COVID 19 from the statement. We have replaced with pandemic.**

**We have corrected the aim in the abstract to be same with that in the introduction. See page 5 line 103-108**

**Reviewer’s comments**

3. Methods- The authors revealed the study instrument here as structured self-administered questionnaires. A concise detail of the questionnaire items would have enlightened the reader. Would all the respondents have understood the items? An interviewer-administered questionnaire could have been a better approach.

**Authors response**

**We used interviewer-administered questionnaire. We have added this in methodology. See page 7 line 148-150**

**Reviewer’s comments**

4. Results- The ‘practice’ aspect of this study namely ‘the wearing of face mask in children’ was based on proxy-report and not observational. Thus, the information provided by these mothers on the proportion of their children who did wear face mask may not be reliable.

**Authors response**

**The information given by the mother was supported with what was observed as the mothers were present with the children during the study. See page 6 line 130-131**

**Reviewer’s comments**

This underscores the fact that an observational study of mother-child dyads would have been better. For instance, the authors reported that children aged 8 days to 1 year wore face masks. The practice is even contradictory to the reported WHO recommendation in children.

**Authors response**

**This was what was observed and reported. Another aspect of the study was providing information to the mothers on the correct use of facemasks. In fact, the finding of children aged 8 days to 1 year wearing face mask is an eye opener and will help alert the government to strengthen policy on acceptable age of wearing face mask and organize program geared on health education. See page 15 line 284-292**

**Reviewer’s comments**

The authors reported some socio-demographic variables as predictors of the practice of wearing face mask in children. Curiously, they were silent on the role of knowledge and perception. Maternal knowledge and perception ought to influence their practice of using face mask on their children

**Authors response**

**This has been included. Thanks for the observation. How the perception of COVID-19 by the mothers was assessed has also been included. See table 2 and 3**
**Reviewer’s comments**

5. Discussion- The content and flow of this section should be improved. The authors did not highlight any limitations of the current study. The conclusion was not precise enough but was too concise to have conveyed the message of the study.

**Authors response**

**We have added limitation to the study, broadened the conclusion and we have mentioned recommendation for policies and program. See page17 line 329-342**

Once again, the authors thank you, the editorial team and the reviewers most sincerely for your critical review and suggestions which have improved the write up of this manuscript.

We hope this revised version of the manuscript will now be suitable for publication in your Journal.

Thank you Sir,

**Dr. Chinawa Josephat**

*Corresponding Author*
