# Supplementary material for: Maternal perception of masking in children as a preventive strategy for COVID-19 in Nigeria: A multicentre study
Source: PLoS One. 2020 Nov 19;15(11):e0242650. doi: 10.1371/journal.pone.0242650 (PMC7676872; doi:10.1371/journal.pone.0242650)
Supplement: Attachment — Submitted filename: REVIEWERS COMMENTS FOR REVISED MS (PLoS ONE).docx [file pone.0242650.s005.docx]

REVIEWER’S COMMENTS ON REVISED MANUSCRIPT (PONE-D-20-19085R)

TITLE- Wearing of face mask by children and associated maternal factors; in the prevention of

COVID 19: A multicentre study

**General comments**

Although the authors tried to address some of the issues pointed out in my previous review, the answers are not yet satisfactory. The revised manuscript still requires some modifications to improve its quality as there are obvious inconsistencies. The casuistry adopted by the authors in some of their responses has not been able to convince me on a number of specific issues which I have highlighted below

**Specific issues and comments**

1. Title: The current version of the title appears tenuous and does not reflect the message of the paper. Undoubtedly, the study was a KAP study on maternal perception of a preventive strategy for COVID-19 and not an observational study. I therefore suggest the authors modify the title thus- ‘Maternal perception of masking in children as a preventive strategy for COVID-19: a multicentre study’
2. Abstract: The objective (or aim) as currently stated can be modified to read- ‘The study aimed to ascertain the perception of mothers on masking in children as a preventive strategy for COVID-19.’ The summary of the Results here should be re-written for clarity. For instance, the statement- ‘A minor proportion of the children, 44.7% do wear face mask’- appears untenable in the context of this study. I presume the authors meant- ‘Minority (44.7%) of the mothers perceive masking in children as an appropriate measure for the prevention of COVID-19’. The succeeding sentence- ‘The major reasons for the children not wearing face mask include perceived difficulty in breathing, 38.2% and that the child pulls it off, 29.3%.’- is suggested to read- ‘The major reasons given by the mothers for the inappropriateness of face mask in children include perceived difficulty in breathing (38.5%) and the child’s readiness to pull it off (29.3%).’Again, one wonders how the authors arrived at the inferential statistics about the effect of maternal age, children’s age paternal educational status and maternal perception of COVID-19 on masking in children. The impression created here is that the authors actually observed masking in the children of these mothers. On the contrary, I suspect the authors merely evaluated the perception of selected mothers on the subject of masking in children as a preventive strategy for COVID-19. The conclusion that ‘The perception of mothers on the children wearing face mask is low’ should rather read-‘Maternal perception of masking in children as an appropriate strategy for preventing COVID-19 is adjudged low in this study. Right perception is significantly enhanced by maternal educational status etc.….’
3. Introduction: The opening sentence as corrected by the authors- ‘Corona Virus pandemic has now attained community infection and is now very common in families’- still appears ambiguous. What do they mean by the pandemic now attaining community infection? The statement after the study aim which reads- ‘It will also form the first data base on this topic in this region’- is considered a redundant statement and should be deleted.
4. Materials and Methods: What do the authors mean by ‘The outcome variables in this study are the level of knowledge and perception of mothers on children wearing face mask’? (Lines 152-153). Nevertheless, this statement gives away the crux of the study as maternal knowledge/perception on masking in children as a preventive strategy for COVID-19. Thus, the authors should not be reporting about proportions of children who do wear face mask but rather on proportions of mothers with varying perceptions/knowledge on masking in children as a pandemic antidote. Again, this statement in lines 154 to 155 – ‘We used age, marital status, educational level, parity, husband’s educational level and occupation as independent variables’- is unclear. Do these variables-age, marital status and educational level-refer to the mothers?
5. Results: The authors in trying to justify the fact that they observed the children of the mothers wearing face mask stated ‘The information given by the mother was supported with what was observed as the mothers were present with the children during the study. See page 6 line 130-131’. Did the authors study mother-child dyads? It was not stated in this study. What form of face masks were worn by the children that were neonates and infants? Do the authors have customized face masks for such age groups in their region? The WHO dissuades the use of face masks in children less than 2 years. Recently, the age bar has been raised to 12 years.
6. Discussion: The revised discussion is not yet robust enough. Several syntax errors and redundant sentences precluded its clarity. For instance, the opening sentence-‘This study has gone a long way to show the importance of prevention of COVID 19 in children by wearing of face mask’ is ambiguous. Several of such statements are littered through this section. Even the study limitation is unrealistic. How could the authors not have observed if the mothers wore face masks since they claimed they observed masking in their children? If it was an oversight, it further confirms my belief that the study was fundamentally conducted on evaluating maternal perception/knowledge on masking in children.
