# Supplementary material for: Maternal perception of masking in children as a preventive strategy for COVID-19 in Nigeria: A multicentre study
Source: PLoS One. 2020 Nov 19;15(11):e0242650. doi: 10.1371/journal.pone.0242650 (PMC7676872; doi:10.1371/journal.pone.0242650)
Supplement: Attachment — Submitted filename: SECOND REVIEW OF MANUSCRIPT- FOR PLoS.docx [file pone.0242650.s007.docx]

**Re- Maternal perception of masking in children as a preventive strategy for COVID-19 in**

**Nigeria: a multicentre study.**

REVIEWER’S COMMENTS

The authors have done pretty well in addressing the concerns raised in the previous review. However, there are still some specific issues that need their attention to further improve this manuscript

1. Under the ABSTRACT, some statements need correction. I suggest these lines should read- ‘The frequent reasons given by majority (55.3%) of the mothers for the inappropriateness of face mask in children…….’ ‘A significantly higher proportion (64.2%) of the children…..would ***wear face*** masks when compared with 31.7% of those whose mothers were < 30 years of age (x^2^ =28.632, p<0.001).’ Similarly, a significantly higher proportion (51.0%) of the children who were more than one year of age ***would wear*** face mask when compared with 20.5% of those aged eight days to one year (x^2^=19.441, p<0.001).’
2. Lines 118-119 (Under INTRODUCTION), the study aim should be written as it appears in the ABSTRACT.
3. Under RESULTS, lines 191-194 should reflect what is in the Abstract. As currently written, the message is inconsistent with that in the Abstract
4. Under DISCUSSION, lines 230-231 read ‘Children less than 2 years may not benefit….may rather be in danger.’ The authors should add ‘….may rather be in danger of suffocation’. Line 252 should be re-written thus- ‘Few mothers in this study…’ Lines 274-276- In line with my previous suggestions, ‘wear face masks’ should rather read ‘would wear face masks’ to be in keeping with the study title of maternal perception of masking in children. Similarly, lines 295-298 should be corrected accordingly. For line 313- delete this redundant statement-‘These assertion is also upheld in our study.’
5. The study limitation as currently stated is ambiguous. The authors should rather state that they did not evaluate the concordance of maternal perception of masking with actual wearing of face mask in their children.
6. The authors should avail themselves of the English language copy-editing services to improve the quality of their English lexicon
