# Supplementary material for: Maternal perception of masking in children as a preventive strategy for COVID-19 in Nigeria: A multicentre study
Source: PLoS One. 2020 Nov 19;15(11):e0242650. doi: 10.1371/journal.pone.0242650 (PMC7676872; doi:10.1371/journal.pone.0242650)
Supplement: Attachment — Submitted filename: THE FINALLY POINT BY POINT ORIGINAL- FOR PLoS.docx [file pone.0242650.s008.docx]

**Re- Maternal perception of masking in children as a preventive strategy for COVID-19 in**

**Nigeria: a multicentre study.**

REVIEWER’S COMMENTS

Review Comments to the Author

Reviewer #1: A minor proportion of the children, 44.7% wore face 194 masks which were all home-made.
According to Table 1 this should be 43.5% 44.7% are females

**This is now corrected. See Page 9,line 197-198**

230. Children less than 2 years may not 231 benefit from wearing face mask but may rather be in danger. –Please clarify this statement. I am assuming that you are suggesting that wearing a mask maybe more high-risk for them

**We have modified the statement. See Page 13 line 238**

The authors have done pretty well in addressing the concerns raised in the previous review. However, there are still some specific issues that need their attention to further improve this manuscript

1. Under the ABSTRACT, some statements need correction. I suggest these lines should read- ‘The frequent reasons given by majority (55.3%) of the mothers for the inappropriateness of face mask in children…….’ ‘A significantly higher proportion (64.2%) of the children…..would ***wear face*** masks when compared with 31.7% of those whose mothers were < 30 years of age (x^2^ =28.632, p<0.001).’ Similarly, a significantly higher proportion (51.0%) of the children who were more than one year of age ***would wear*** face mask when compared with 20.5% of those aged eight days to one year (x^2^=19.441, p<0.001).’

**This has been corrected. See Page 2 line 37-45**

2. Lines 118-119 (Under INTRODUCTION), the study aim should be written as it appears in the ABSTRACT.

**We have written the study aim as it appeared in the abstract. See Page 6 line 119-120**

3. Under RESULTS, lines 191-194 should reflect what is in the Abstract. As currently written, the message is inconsistent with that in the Abstract.

We have reflected statements in results to be in keeping with that in abstract.

**See page 9 line 193-198**

Under DISCUSSION, lines 230-231 read ‘Children less than 2 years may not benefit….may rather be in danger.’ The authors should add ‘….may rather be in danger of suffocation’.

**We have added the phrase may rather be at risk of suffocation’. See Page 13 line 238**

4. Line 252 should be re-written thus- ‘Few mothers in this study…’

**This has been added. See Page 14 line 260**

5. Lines 274-276- In line with my previous suggestions, ‘wear face masks’ should rather read ‘would wear face masks’ to be in keeping with the study title of maternal perception of masking in children.

**This has been effected. See Page 15 Line 282-283**

6. Similarly, lines 295-298 should be corrected accordingly.

**This has been corrected. See Page 15 Line 299**

7. For line 313- delete this redundant statement-‘These assertion is also upheld in our study.’

**This has been deleted**

8. The study limitation as currently stated is ambiguous. The authors should rather state that they did not evaluate the concordance of maternal perception of masking with actual wearing of face mask in their children.

**We have added this in limitation. See Page 18 line 350-351**
