# Supplementary material for: Maternal perception of masking in children as a preventive strategy for COVID-19 in Nigeria: A multicentre study
Source: PLoS One. 2020 Nov 19;15(11):e0242650. doi: 10.1371/journal.pone.0242650 (PMC7676872; doi:10.1371/journal.pone.0242650)
Supplement: Attachment — Submitted filename: MOST ZENNIT FACE MASK POINT BY POINT.docx [file pone.0242650.s009.docx]

Reviewer #1: Thank you for addressing the comments. Please clarify the following
Table 1 shows the wearing of face mask by the children. Minority (44.7%) of the mothers 193 perceived masking in children as an appropriate measure for the prevention of COVID-19. The194 frequent reasons given by majority (55.3%) of the mothers for the inappropriateness of face
195 mask in children included perceived difficulty in breathing (38.5%) and the child’s readiness 196 to pull it off (29.3%)

**This statement has been expunged and the section rewritten. See page 9 line 192-195**
In table1- 44.7 and 55.3 refers to gender - male and female. Please clarify if it is for mothers? the table is titles Child wearing a mask so its very confusing.

**This has been corrected. See page 9 line 192. See also Table one**

Reviewer #2: General comments:
Although the authors have progressively improved the quality of this manuscript by their revisions, I would urge them to be more painstaking in responding to the specific issues of concern previously raised. This will not only eliminate inconsistencies within the manuscript but also improve the lexicon of their written English.
Specific comments:
1. Abstract- In line 41, the repeated figure (64.2%) should be deleted.
**This has been deleted.**

2. In my previous reviews, I pointed out that the manuscript has grammar and syntax errors. I suggested an English editing to be done. However, it appears the authors have disregarded this suggestion. This is evident in some errors noted as follows: ‘was’ used instead of ‘were’ (Line 77), ‘is’ used instead of ‘are’ (Line 78), ‘In China’ instead of ‘in China’ (Line 90) and ‘intensified program’ used instead of ‘intensified programs’, etc.

**We have sent the paper to Grammarly for English editing. We believe that the paper looks better than the previous one. The corrected syntax is as highlighted. See page 4 and 5**

Furthermore, I guess the statement in line 145 should read ‘we did not focus on mother-child dyads while assessing maternal perception and the practice of wearing face masks, though.’

**This has been corrected. See page 7 line 146-148**

In fact, this assertion throws up an inconsistency with lines 178-179 where the authors stated that a ‘Yes’ answer on wearing face mask was confirmed by their observation of masking in children during data collection. How could the authors not have focused on mother-child pairs in the study when they reported masking in both the mothers and their children

**Though we reported masking in both mothers and their children, but unfortunately, we did not ascertain the practice of masking on mother-child pair as this was not factored in the questionnaire. We are sorry for this.**

This again negates the study limitation in lines 350-351 which states ‘We did not evaluate the concordance of maternal perception of masking with actual wearing of face mask in their children.

**The study limitation now reads “We did not ascertain actual practice of masking on mother-child pair”. See page 17 line 343**

’ Let the authors correct this inconsistency
**We have tried to correct these inconsistencies**

3. Finally, I urge the authors to subject the manuscript to English editing. I suggest they could use the ‘grammarly’ software if they cannot utilize the available English editing services

**We have subjected the article to English editing using Grammarly.**
